# Advances in Tissue Engineering and Biomaterials for Minimizing Wound Scarring: Current Status and Future Challenges

**DOI:** 10.3390/jfb17010003

**Published:** 2025-12-19

**Authors:** Tingting Weng, Lulu Zhang, Yuxin Liu, Xiong Zhao, Xiaojie Yue

**Affiliations:** 1 Department of Burn and Plastic Surgery, Children’s Hospital, Zhejiang University School of Medicine, Hangzhou 310052, China; 11918370@zju.edu.cn (T.W.); 6513029@zju.edu.cn (L.Z.); 6516036@zju.edu.cn (Y.L.); 6196001@zju.edu.cn (X.Z.); 2 National Clinical Research Center for Child Health, National Children’s Regional Medical Center, Hangzhou 310052, China

**Keywords:** tissue engineering, biomaterials, bioactive factors, scarless healing, wound healing

## Abstract

Current wound-healing strategies still fall short in achieving scarless regeneration. Adult wounds often heal in the form of scars, leading to functional impairment and aesthetic concerns. In contrast, fetal wounds exhibit a remarkable capacity for scarless regeneration due to their unique immune environment and regenerative cellular responses. This review systematically elucidates the key mechanisms underlying scarring and contrasts them with the hallmarks of fetal scarless healing. We then comprehensively explore engineering strategies to minimize scar formation in adults, with a focus on the combined application of regenerative biomaterials, bioactive factors, and cell therapies. Particularly emphasis is placed on novel intelligent biomaterials for reprogramming wound immune microenvironment and precise delivery strategies of “spatiotemporal sequential” or “on-demand”. These innovations signify a shift toward precise and controlled therapeutic intervention, offering new pathways for skin regeneration that enhances both functionality and aesthetics.

## 1. Introduction

As the body’s primary interface with the external world, the skin is the first to suffer from injury, and skin wounds often heal in the form of scars [1]. Annually, mechanical injuries, burns, chronic ulcers, and traumatic events lead to cutaneous scarring for millions of individuals globally. Pathological scars can subsequently give rise to significant clinical challenges, including functional deficits, restricted mobility, aesthetic concerns, and profound psychological distress [2,3,4]. Therefore, the healing method for scars has become the focus of many scholars. However, its therapeutic effect remains unsatisfactory.

Scar formation is an inevitable outcome of wound healing. Wound healing is a complex process involving inflammatory cells, repair cells, extracellular matrix (ECM), and cytokines, which are highly coordinated [5]. The healing process typically unfolds through the following three sequential phases: initial inflammation, subsequent proliferation, and final remodeling [6]. In the inflammation stage, the injured area was swollen, tissue exudate increased and inflammatory cell infiltration was prominent. In the proliferative phase, many vascular endothelial cells participated in neovascularization, and new capillaries and proliferating fibroblasts formed fresh granulation tissue. During this period, fibroblasts secreted various proteases, including matrix metalloproteases (MMPs), for degrading the temporary matrix, and secreting ECM such as collagen to fill the wound, providing a support structure for cell adhesion, migration, proliferation, growth and differentiation during wound healing. During the remodeling period, the nascent ECM, collagen fibers, and elastic fibers are rearranged under the action of physical tension and cytokines. A pivotal event in this process is the differentiation of fibroblasts into myofibroblasts, which can express alpha-smooth muscle actin (α-SMA) and facilitate wound contraction [7]. Research has established that the three phases of wound healing are closely related to scar formation, and largely determine whether the outcome is regenerative (scarless) or fibrotic (scarred) [4,8].

Pathological scarring primarily results from the excessive and disorganized deposition of connective tissue, mainly collagen, during repair. The causes of scar formation are highly complex, including dermal defect, inflammatory reaction, excessive secretion and deposition of ECM, abnormal tissue remodeling, etc. Scars can be divided into physiological scars and pathological scars. Physiological scars generally do not require treatment. Pathological scars mainly include keloid and hypertrophic scars (HTS), and their pathogenesis remains unclear. Current clinical interventions include surgical resection, laser therapy, radiation, cryotherapy, pressure therapy, and intralesional corticosteroid injections, among others (see Table 1) [9,10]. However, these treatments cannot remove the pathological scar completely. Scarless repair after skin injury is an ideal healing model based on physiological and psychological needs. By serving as a dermal substitute, biomaterials can mitigate scar formation during wound repair by regulating wound microenvironment inflammation and cell behavior. Herein, this review initially provides the mechanisms underlying scar formation. Subsequently, it contrasts the distinct healing processes observed in fetal adult. The core of the discussion focuses on the latest advancements in engineered strategies for scarless repair, delving into the applications of biomaterials, bioactive factors, and stem cells.

## 2. Scar Healing Mechanism

**Figure 1:**
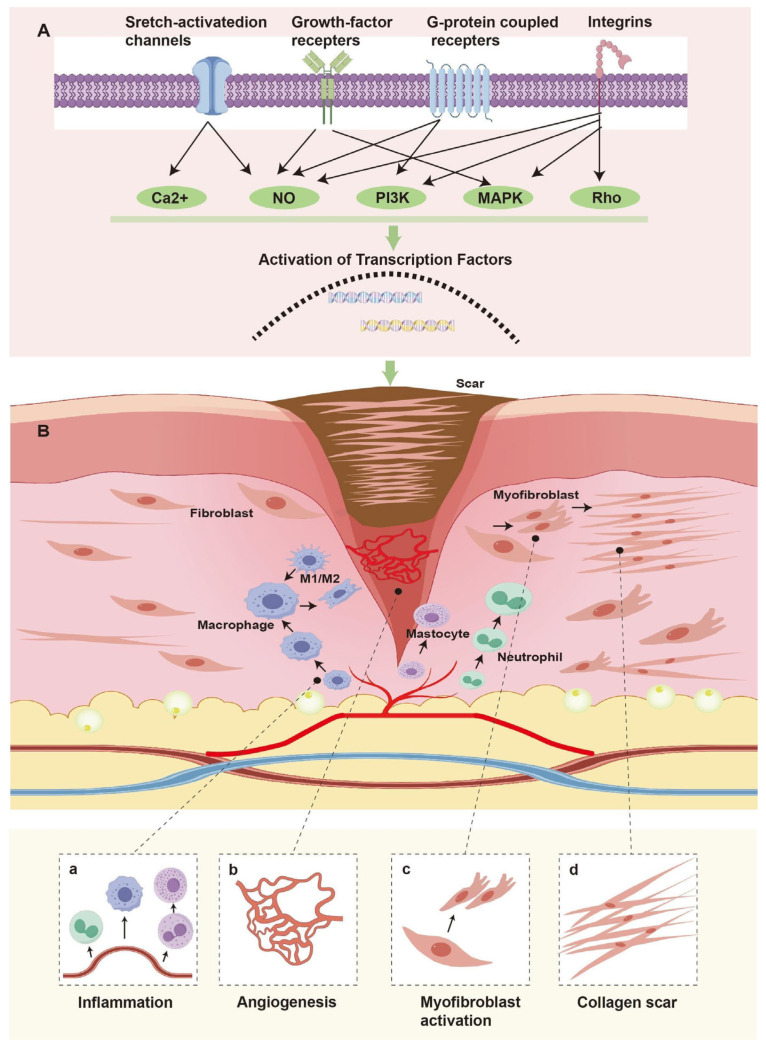
Schematic representation of scar healing mechanism. (**A**) After skin injury, various stimuli activate intracellular scar formation related signal pathway. The signal cascades activate transcription factors that translocate into the nucleus and activate mechanically regulated genes. (**B**) Schematic diagram of wound scar healing. (**a**) Neutrophils, macrophages and mastocytes migrate into the wound by extravasation from nearby vessels and cause excessive inflammation. (**b**) Excessive angiogenesis in wound scar healing. (**c**) Fibroblasts activate into myofibroblasts. (**d**) ECM remodelling to form scars. Figure 1 was drawn by authors in Adobe Illustrator 2023.

Wound repair constitutes a sophisticated and multi-stage physiological progression, characterized by granulation tissue development, diverse tissues regeneration, and eventual scar maturation. At present, researchers are working hard to deeply analyze the mechanism of wound scar formation (see Table 2) and actively explore effective prevention and treatment targets, and significant progress has been made in related fields (as summarized in Figure 1).

The inflammatory response is an important stage in skin wound healing. Moderate inflammation is necessary to remove the necrotic tissue. However, persistent necrotic tissue leads to infection, delayed wound healing, and significantly increases the risk of pathological scarring. The involved signaling pathways include transforming growth factor-β (TGF-β)/Smads signaling pathway, Rho/ROCK signaling pathway, etc. Studies have revealed that factors that regulate the synthesis or secretion of TGF-β can affect the formation of scars through TGF-β/Smads signaling pathway [11,12,13]. The roles of TGF-β/Smads signaling pathway in scar formation mainly include: promoting the proliferation and migration of fibroblasts (Fbs), collagen synthesis and secretion, and inhibiting the degradation of type I collagen [14]. Rho protein is a small-molecule G protein, including RhoA, RhoB, RhoC, and RhoC, and it plays a significant role in scars. Activated RhoC can phosphorylate ROCK, which in turn promotes Fb proliferation and matrix protein synthesis, resulting in massive deposition of ECM proteins and promotion of scar formation [15].

Tissue repair is primarily accomplished by the activities of the Fbs. The signaling pathways related to Fb activity include the p38MAPK, ERK and integrin signaling pathway, etc. The p38MAPK and TGF-β/Smads signaling pathway have mutual regulation [16]. The current research showed that p38MAPK inhibitor could not only effectively inhibit the synthesis of TGF-β1 and the secretion of ECM, but also inhibit the contraction of smooth muscle actin, especially in the wound healing with tension [17]. Key factors such as connective tissue growth factor (CTGF) and epidermal growth factor (EGF) serve as primary activators of the ERK signaling pathway. Once activated, this pathway orchestrates fibroblast proliferation and stimulates collagen synthesis and secretion, which are central events in scar formation [18]. The increase of wound tension is a critical activator of the integrin signaling pathway. This mechanical force directly activates integrin receptors, which function as key transducers, transforming extracellular physical cues into chemical signals within the cell [19]. This mechanotransduction subsequently promotes a profibrotic response characterized by fibroblast proliferation, differentiation, and enhanced collagen secretion [20].

Furthermore, angiogenesis during wound healing is closely related to scar formation. Massive angiogenesis has long been considered beneficial to wound healing [21]. However, this view has recently been questioned [22,23]. Potential mechanisms of angiogenesis affecting scar formation included: (1) the presence of a large number of angiogenic growth factors (VEGF, PDGF, FGF, etc.) in the wound-activated PI3K signaling pathway and promoted scar formation. This pathway mainly mediated the neogenesis of vascular endothelial cells and is one of the important targets for IL-10 to inhibit scar formation [24]. (2) In the wound healing process, most new blood vessels had high permeability, which caused local tissue edema and increased wound tension, thus activating the integrin signaling pathway and forming scars [25]. In conclusion, while we should be concerned about the delayed wound healing caused by insufficient blood supply, we should also be aware that inhibiting excessive angiogenesis may potentially lead to scar prevention and treatment.

**Table 2:** Key signaling pathways involved in scar formation.

Pathway	Primary Activators	Core Pro-Fibrotic Functions in Scarring	References
TGF-β/Smad	Excessive inflammation Increased wound tension	Promotes fibroblast proliferation/migration, stimulates collagen synthesis, inhibits collagen degradation.	[11,12,13,14]
Rho/ROCK	TGF-β	Drives fibroblast proliferation and matrix protein synthesis, leading to excessive ECM deposition.	[15]
p38MAPK	TGF-β1	Mediates TGF-β1 secretion and ECM synthesis, critical in tension-associated scarring.	[16,17]
ERK	CTGF, EGF	Coordinate the proliferation of fibroblasts, promote the synthesis of collagen.	[18]
Integrin	Increased wound tension (Mechanotransduction)	Transduces mechanical force into chemical signals, promote fibroblast activation and fibrosis.	[19,20]
PI3K	Angiogenic factors (VEGF, PDGF, FGF)	Mediates pathological angiogenesis, linked to vascular overgrowth in scars.	[24]

## 3. Characteristics of Fetal Scarless Healing

In adults, when the skin damage reaches a certain level, granulation tissue fills the defect and then gradually fibrosis to form a scar. In contrast, scarless healing of fetal wounds occurs only during prenatal development [26]. Some possible influencing factors have gradually been discovered as research has progressed. However, the specific mechanism remains unclear.

Four major differences were discovered in wound healing in fetuses and adults: (1) Inflammatory reaction. A hallmark of fetal healing is a markedly reduced inflammatory response. Unlike adult wounds, which exhibit robust infiltration of neutrophils and macrophages, fetal wounds show minimal to no inflammatory cell recruitment [27]. Martin et al. observed that adult PU.1 knockout mice with low inflammatory response levels can still achieve scarless healing without the involvement of macrophages and neutrophils [28]. In summary, the lack of inflammatory cells reduces the level of inflammatory factors, reduces the damage to the tissues around the wound, and provides an environment for scarless wound healing [29]. (2) Differences in Fbs. Fibroblast heterogeneity plays a decisive role. During early embryogenesis, the majority of dermal fibroblasts belong to the anti-fibrotic, En1-negative (ENF) lineage. However, a pronounced shift occurs postnatally, with pro-fibrotic En1-positive (EPF) cells becoming the predominant population [30]. Lineage-tracing studies confirmed that EPFs possessed a consistent capacity to form scars both at embryonic and post-natal stages, suggesting that the underlying mechanism of transition from scarless fetal wound healing to more fibrotic repair in adult skin is reflected in the alteration of En1-lineage populations during development [31]. Notably, transplantation of ENFs into adult wounds can mitigate scarring, in part by promoting angiogenesis and improving communication between repair cells [32]. (3) Regenerative growth factor profile. Studies have shown that the expressions of TGF-β1 and TGF-β2 in the process of scar healing in adult wounds were significantly higher than those in fetal scarless healing wounds [33]. In contrast, TGF-β3 is highly expressed in fetal keratinocytes and fibroblasts, but expresses at low levels or absent in adult wounds [34,35]. And the activity of MMPs, crucial for ECM remodeling, is significantly elevated in fetal wounds compared to adults [36]. Moreover, platelet-derived growth factor (PDGF) was expressed at high levels in adult wounds but not in fetal wounds [37]. (4) Unique ECM composition. Fetal wounds are characterized by rapid and sustained deposition of hyaluronic acid (HA), which persists significantly longer than in adult wounds and promotes a regenerative healing by modulating collagen expression and TGF-β3 signaling. Locono et al. confirmed that an appropriate increase in HA could significantly reduce the scars formed during wound healing [38]. The collagen ratio is also distinct, with fetal wounds depositing a higher proportion of type III to type I collagen, contributing to a more organized and less fibrotic matrix [39,40].

Fetal scarless wound healing is a common phenomenon in mammals, and it is different from adult wound healing in many aspects, suggesting that these different factors may be related to fetal scarless wound healing (see Table 3). The specific mechanism needs further experimental verification. However, artificially changing these factors in the experiment can successfully reverse scar healing to scarless healing, providing a new idea for clinical scarless healing.

## 4. Biomaterials for Scarless Healing

**Figure 2:**
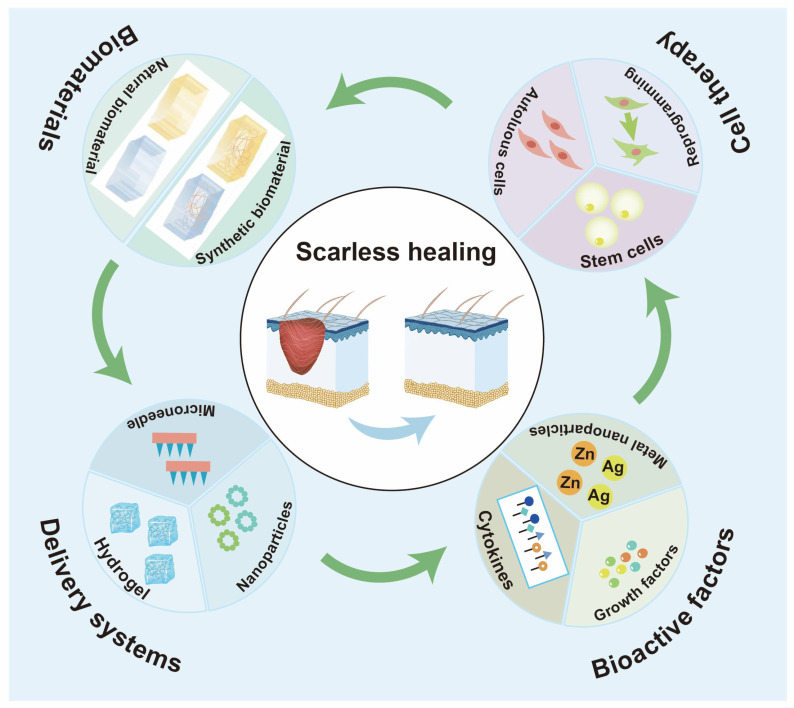
Schematic representation of tissue engineering and biomaterials for wound scarless healing. Figure 2 was drawn by authors in Adobe Illustrator 2023.

Currently, the treatment of scars cannot fully meet the needs of people, mainly manifested as difficulty in completely removing all the pathological scars. Therefore, scars are still challenging in the cosmetic and surgical area [41]. However, advancements in tissue engineering are bringing more alternative treatments closer to becoming a therapeutic reality. Furthermore, the functional combination of these biomaterials with various bioactive factors and cells enables a precisely regulated approach to scar treatment. Hence, we focus here on the engineering methods of scarless wound healing, using biomaterials, different bioactive factor and stem cells for scarless healing (Figure 2). Accordingly, the central focus of this discussion is on engineering strategies for achieving scarless repair that utilize these combined systems.

### 4.1. Pure Biomaterial

#### 4.1.1. Natural Polymer Materials

Several natural polymer materials, such as collagen, gelatin, fibrin and HA, have been used for wound repair to reduce scar formation. HA plays an important role in tissue regeneration and wound healing. Fetal tissues containing high concentrations of HA heal quickly and leave no scars. Hu et al. [42] reported that grafting with HA chains significantly accelerated wound closure and diminished scar size. The therapeutic outcome associated with reduced TGF-β1 levels within the wound site. A separate investigation in rabbit models provided that injectable HA hydrogels similarly enhanced healing and mitigated scar formation. The results showed that HA hydrogels could improve wound viscoelasticity by reducing fibronectin levels, TGF-β1, procollagen I, and HA synthase, thereby increasing fibronectin levels and healing properties of HA in regenerated tissues [43].

Bacterial cellulose (BC), a natural polysaccharide produced by specific bacteria, exhibits notable biological properties including high biocompatibility, biodegradability, and bioactivity [44]. In a clinical study involving patients with second-degree facial burns, Czaja et al. demonstrated that BC application significantly accelerated wound closure, alleviated pain, and minimized scar tissue formation, while also facilitating the removal of necrotic debris and enhancing re-epithelialization compared to standard care [45]. Similarly, collagen possesses excellent biocompatibility and supports key cellular processes such as adhesion, proliferation, and migration [46]. Rastogi et al. [47] assessed a collagen membrane as a biodegradable dressing for surgical wounds in the oral mucosa. Their findings indicated that the membrane served as a superior alternative to existing graft materials. By mitigating infection during initial healing and curbing excessive granulation tissue formation, it resulted in diminished scarring and a short healing duration. Silk fibroin (SF) produced by silk worms has been recognized as a promising scaffold material because of its biocompatibility, biodegradability, high mechanical strength, and flexibility. SF scaffolds can support cell adhesion, proliferation, and differentiation in vitro, and promote tissue repair in vivo [48]. Zhang et al. [49] pioneered a minimally invasive and practical strategy for achieving scarless tissue regeneration using a SF microneedle patch. They discovered that merely modulating the physical dimensions and density of the microneedles markedly lowered the scar elevation index in a rabbit ear HTS model. Concurrently, the treatment restored the ultimate tensile strength to levels comparable to normal skin. In summary, the physical intervention afforded by microneedle system represents a promising mechano-therapeutic strategy for scarless wound repair. Gelatin, valued for its biocompatibility and biodegradability, is extensively employed in the fabrication of wound dressings [50]. Leveraging this material, Shan et al. developed a SF/Gelatin electrospun nanofibrous dressing for treating deep partial-thickness burn wounds. The application of this dressing markedly accelerated healing and suppressed scar formation in vivo [51].

Acellular dermal matrices (ADM) are biomaterials fabricated by subjecting the dermis to rigorous physical and chemical decellularization processes. This treatment eliminates all cellular constituents that could trigger host immune rejection, while preserving the native ECM architecture and key components, including collagen types I and III, elastin, proteoglycan, etc. [52]. Therefore, ADM exhibits high biocompatibility and provides abundant cell growth and differentiation signals, which are conducive to cell proliferation, differentiation and tissue repair. Among them, xenogeneic ADM dressing, as a temporary wound cover, has become a commonly used material in clinical practice and has received good results. The wound covered with xenogeneic ADM dressings could effectively protect the wound surface, reduce exudation, prevent infection, promote wound healing, reduce scar formation, and make the healed skin close to the normal skin as much as possible [53]. Feng et al. [54,55] found that porcine ADM dressing could reduce exudate, relieve pain and inflammation, and inhibit scar formation in treating second-degree burn wounds. Chen et al. [56] used porcine ADM with autogenous split-thickness skin transplantation and obtained good results. The long-term follow-up revealed that the combination of xenogeneic ADM and a split-thickness autograft provided durable restoration of full-thickness skin injuries. Critically, this strategy not only suppressed excessive scar formation but also demonstrated long-term tolerability without eliciting an immune rejection response.

#### 4.1.2. Synthetic Polymer Materials

In addition to natural biomaterials, many synthetic materials have been used for scarless healing of wounds. For example, Lorden et al. [57]. studied an electrospun micro-fibrous scaffold fabricated from the copolymer poly(L-lactide-co-ε-capro-lactone) (PLCL). In vitro analysis demonstrated that PLCL scaffolds decreased myofibroblast formation.

Animal experiments found a more pronounced contraction of HTS in animals receiving the standard treatment (Integra) compared to those grafted with collagen-coated-PLCL (ccPLCL) scaffolds. In a related investigation, Lorden et al. [58] assessed the effect of the biostable polyurethane scaffold on HTS progression. Their findings indicated that biostable polyurethane scaffolds effectively mitigated the HTS contraction including decreased scar tissue stiffness and limited contraction in vivo. Collectively, these results imply that the sustained presence of a scaffold throughout the wound remodeling phase offers a promising and clinically translatable strategy for controlling HTS contraction. Kim et al. [59] developed electrospun nanofibrous wound dressings from a biodegradable polyester, poly (3-hydroxybutyrate-co-3-hydroxyvalerate) (PHBV). The results demonstrated that these PHBV meshes effectively attenuate pathological scarring, a beneficial outcome attributed to the suppression of myofibroblast differentiation, thereby highlighting their therapeutic potential as advanced wound dressings. Lu et al. synthesized ROS-scavenging hydrogel. In an in vivo model of full-thickness skin defects infected with MRSA, application of this ROS-scavenging hydrogel significantly promoted scarless repair. The therapeutic effect was achieved through a multifunctional mechanism involving quorum-sensing disruption, potent bactericidal activity, inflammation suppression, and angiogenesis promotion. This result offered a new insight for achieving scarless healing in clinically challenging MRSA-infected wounds [60].

### 4.2. Biomaterials Incorporated with Bioactive Factors

The field of tissue engineering is progressively paving the way for novel therapeutic interventions. A key strategy involves the functionalization of engineered biomaterials with specific bioactive factors, which enables a highly regulated approach to scar management.

At all stages of wound healing, growth factors promote collagen synthesis and deposition, angiogenesis, wound re-epithelization, and inhibiting scar formation. In recent years, various of delivery systems have been developed to assure better stability and controlled release of growth factors to achieve of scarless healing of wounds [61,62]. At the wound remodeling stage, basic fibroblast growth factor (bFGF) reduces collagen synthesis by increasing the degradation of procollagen mRNA and inhibiting mRNA transcription. In addition, bFGF can increase MMP to degrade collagen and inhibit the terminal differentiation of myofibroblasts by reducing scar formation [63]. Xu et al. [64] engineered a novel liposomal system featuring a hydrogel core of SF (SF-LIP). This SF-LIP system achieved high-efficiency encapsulation of bFGF and conferred a threefold enhancement in its stability within wound exudate compared to the free form. bFGF-loaded SF-LIP significantly accelerated the closure of wounds in mice and concurrently attenuated scar tissue formation. EGF also reduces the formation of scars by inhibiting the inflammatory response and reducing the expression of TGF-β1 during the wound remodeling stage [65]. Drawing inspiration from the privileged scarless healing of oral mucosa, Kong et al. [66]. engineered a multifunctional hydrogel designed to recapitulate key aspects of the oral mucosal wound microenvironment. Their system strategically encapsulated EGF within layered self-assembled microcapsules while incorporating bFGF directly into the hydrogel matrix, thereby achieving a spatiotemporally differential release profile that mimics natural healing dynamics. They concluded that this comprehensive strategy, which orchestrates the sequential presentation of growth factors while maintaining a sterile, moist milieu, is essential for achieving rapid and scarless skin regeneration.

Interleukin-10 (IL-10) and TGF-β3 have recently emerged as prominent cytokines for scar minimization [67]. IL-10 mitigates scarring by curbing autophagy in fibroblasts and abnormal collagen accumulation through integrated STAT3 and AKT-mTOR signaling [68], while the multifunctional TGF-β3 exerts a critical influence on healing dynamics. TGF-β comprises three isoforms, namely, TGF-β1, TGF-β2 and TGF-β3, and the expression levels of each isoform will gradually change in different stages of wound healing. At the wound remodeling stage, TGF-β3 induces decreased fibroblast differentiation levels, resulting in reduced scar formation [69]. Park et al. [70] prepared a polycation-mediated coacervate (Coa)- exogenous GF delivery platform to effectively deliver TGF-β3 and IL-10. The results indicated that the exogenous administration of dual GF via Coa enhanced the proliferation and migration of skin-related cells. Furthermore, gene expression profiles using RT-PCR revealed up-regulation of ECM formation at an early stage of wound healing and down-regulation of scar-related genes at later stages. Therefore, they conclude that exogenous dual GF delivery via the Coa platform effectively augments the quantity and quality of regenerated skin tissues without scar formation.

Recently, more pharmaceutical ingredients combined with biomaterials have been used to prevent and minimize scars. For example, the application of traditional Chinese medicine ingredients have shown promise in suppressing scar formation. Sun et al. fabricated the chitosan-coated hydrophilic PLGA electrospun fibrous membranes loaded with Ginsenoside-Rg3 (Rg3). In animal models, wounds covered with these membranes exhibited complete re-epithelialization and healed 3–4 days faster than controls. The therapeutic efficacy was further demonstrated by a notably lower scar elevation index and improved histological outcomes at the 28-day endpoint, underscoring the potential of Rg3 in anti-fibrotic treatment [71].

Despite the considerable clinical potential of triamcinolone acetonide (TA) and 5-fluorouracil (5-Fu) in scar management, their divergent metabolic characteristics considerably limit the therapeutic outcome. Yang et al. [72] designed a bilayered dissolving microneedle (BMN) system for co-delivery of TA and 5-Fu (TA-5-Fu-BMN), which was designed to achieve biphasic drug release. The efficacy of this novel system was assessed using a HTS model in rabbit ears. The results demonstrated that treatment with TA-5-Fu-BMN led to a marked suppression of aberrant fibroblast proliferation and a significant reduction in collagen deposition.

Furthermore, metal Zinc nanoparticles are particularly advantageous for wound healing applications due to their low cytotoxicity, antibacterial, and anti-inflammatory activities [73]. In a study, zinc sulfide nanoparticles (ZnS-NPs) were evaluated for their efficacy in both in vitro settings and an in vivo rat full-thickness wound model. Treatment with ZnS-NPs significantly promoted skin regeneration, as evidenced by the induction of skin appendage formation, suppression wound contraction and inhibition subsequent scar tissue formation [74].

To achieve scarless healing of wounds requires the coordinated and orderly interaction of many bioactive factors, so applying a single factor is not the best treatment. In addition, different bioactive factors play an important role in various stages of wound healing. However, the mechanism of each factor and cell remains unknown, necessitating further research

### 4.3. Biomaterials Combined with Cell Therapy

Recently, extensive studies have been conducted on tissue engineering and stem cells application in wound treatment and scar decrease. By integrating cell-based therapeutics with biomaterials, tissue engineering offers a transformative perspective for clinical interventions in wound healing and scar management.

The widespread adoption of cosmetic liposuction provides a plentiful source for harvesting adipose-derived stem cells (ADSCs), which have demonstrated substantial promise in cell-based therapeutics [75,76]. After the occurrence of wounds, the inflammatory response triggers the activation of ADSCs, which initiates the immunoregulatory function, increasing the expressions of prostaglandin -E2 and epoxy enzyme -2, and reduces the pro-fibrosis effect of macrophages. In addition, ADSCs can effectively inhibit the formation of scars by secreting anti-fibrosis factors [77]. There are many clinical and experimental mechanisms concerning treating scars with ADSCs. Some scholars co-culture ADSCs with HTS fibroblasts to observe the inhibitory effect of ADSCs on fibroblasts [78]. In addition, studies have revealed that ADSCs can be injected into the superficial dermis of scars to inhibit scars [79].

A combinatorial strategy utilizing polyhydroxybutyrate-co-hydroxyvalerate (PHBV) scaffolds integrated with ADSCs was developed to provide essential biomechanical and biochemical cues for enhanced wound healing in a full-thickness model. The presence of ADSCs within this construct was found to upregulate the expression of VEGF and bFGF, while also to play a pivotal role in scar modulation by regulating key factors including TGF-β1, α-SMA, and TGF-β3 [80]. Separately, Dong et al. [76] engineered an in-situ forming hydrogel system for ADSCs delivery to treat burn wounds. Bioluminescence imaging of luciferase+ ADSCs indicated that the hydrogel protected the implanted cells from the harmful wound environment in burns. Hydrogel-ADSC treatment significantly enhanced neovascularization, accelerated wound closure and reduced scar formation.

Evidence suggests that mesenchymal stromal cells (MSCs) play a role in wound healing by promoting tissue regeneration and inhibiting the formation of fibrotic tissue. Recent advances in wound therapy frequently employ biomaterials as carriers to deliver mesenchymal stem cells (MSCs) to the wound edges. This strategy has been shown to enhance the rate of wound closure and reduce scarring, which primarily by preserving the bioactivity of MSC-secreted proteins [81]. Li et al. designed a 3D graphene foam (GF) scaffold as a carrier for bone marrow-derived MSCs (BM-MSCs) to promote skin regeneration. Mechanistic investigations revealed that the GF-BM-MSCs scaffolds promoted neovascularization by upregulating VEGF and bFGF. Concurrently, it effectively modulated scar formation through downregulation of profibrotic mediators (TGF-β1 and α-SMA) and upregulation of the anti-fibrotic factor TGF-β3 [82]. Moreover, Sun et al. evaluated the efficacy of a conditioned medium obtained from MSCs (MSC-CM) to treat radiation-induced skin injuries in a rat model. The results demonstrated that MSC-CM treatment significantly enhanced wound closure and improved healing quality. These beneficial effects were attributed to the stimulation of cellular proliferation, the promotion of angiogenesis and the suppression of scar tissue formation [83].

Although many studies have shown that cell therapy combined with biomaterials contributes to the treatment of scars, most of them are in the stage of animal experiments and basic experiments, and there is a lack of a corresponding basis for clinical treatment. Moreover, most reports only cover the early effects of cell therapy combined with biomaterials in wound repair, such as cell proliferation and migration, but ignore the late effects on the wound. As a result, extensive research and clinical application are still required. In the future, ADSCs, MSC, and other cells can be added to find more research targets for anti-scar and pro-repair treatment of wounds.

## 5. Innovative Application of Biomaterials in Scarless Healing

### 5.1. From “Passive Support” to “Active Regulation”: A New Intelligent Biomaterial for Reprogramming Wound Immune Microenvironment

Traditional biomaterials mainly play the role of “passive” physical barriers and scaffolds. In recent years, new biomaterials have emerged in an endless stream, among which the core innovation of the new generation of “active” biomaterials is that they can perceive and intelligently regulate the immune microenvironment of wounds, and then actively guide the regenerative healing [84].

The stiffness of biomaterials is a key signal that determines cell behavior. Biomaterials engineered to mimic the soft compliance of fetal ECM (typically in the range of 0.1–5 kPa) have been shown to promote an anti-inflammatory phenotype in macrophages (M2 polarization) and inhibit the differentiation of fibroblasts into myofibroblasts, thereby reducing scar formation. This is often mediated through mechanotransduction pathways like YAP/TAZ [85,86]. The next frontier lies in creating dynamic matrices whose stiffness can evolve with the healing stages, though this goal remains at the conceptual or early experimental phase.

The homeostasis of the wound microenvironment is fundamental for scarless wound healing. However, the excessive accumulation of TGF-β acts as a primary driver of HTS formation. This dysregulation promotes fibrosis by orchestrating the cell fates and crosstalk among various types of cells, such as macrophages and fibroblasts. An injectable self-assembling LA-peptide hydrogel has been developed. As expected, the LA-peptide hydrogel attenuated M2-like macrophages polarization and reduced fibroblasts activation through the adsorption of TGF-β both in vitro and in vivo, ultimately facilitating scarless healing [87]. Another novel liposomal composite hydrogel is engineered for wound microenvironment remodeling. And this hydrogel promotes macrophage polarization from M1 to M2, effectively regulating inflammatory responses. Additionally, it inhibited the TGF-β/Smads pathway to reduce fibrotic factors, thereby preventing scar formation [88].

In a word, the novel biomaterials actively communicate with the host immune system through their delicate physical and biochemical characteristics. This dialogue facilitates a fundamental shift in the wound healing response, successfully transforming a pro-inflammatory, scar-forming into an anti-inflammatory, pro-regenerative microenvironment, which provides a powerful tool and practical way for the ultimate realization of perfect regeneration without scar.

### 5.2. Beyond Single-Factor Release: Spatio Temporal Sequential or On-Demand Precision Delivery Strategy

Wound healing is a highly complex, multi-stage and orderly biological process. Single and constant bioactive factors factor release cannot reproduce the precise regulation of fetal scarless healing. Here, we innovatively summarize and analyze the advanced delivery system that can imitate the dynamic healing process in vivo and provide the release function of “spatiotemporal sequential” or “on-demand”.

The targeted delivery of bioactive factors presents a central challenge in regenerative medicine. A therapeutic approach designed to emulate the natural wound healing should involve the spatiotemporally patterned release of multiple agents, each administered at a physiological dose and an optimal ratio, rather than relying on a single growth factor [89]. By employing multi-layer electrospun fibers or core-shell microspheres, different functional factors can be selectively loaded into distinct layers. The outer layer can be loaded with anti-inflammatory or antibacterial factors (such as IL-10 and silver ions) at early stage, while the inner layer or core can be loaded with factors (such as VEGF, FGF-2 and TGF-β3) that can promote angiogenesis, matrix remodeling and inhibit scar formation, thus realizing time sequence control [90,91]. The manufacturing process is now relatively mature, making it an ideal candidate material for near-future clinical trials. The primary challenge is optimizing release kinetics to match highly variable human wound timelines.

In recent years, intelligent biomaterials have emerged continuously, which can sense specific signals of wound microenvironment (such as pH value, ROS level, and concentration of specific enzymes (matrix metalloproteinases (MMPs)), and accurately release its therapeutic loads under the trigger of these signals. This realizes “drug delivery on demand” and greatly improves the therapeutic effect and safety [92]. But the transformation challenges for these emerging materials include how to reliably sense triggers in the human wound environment, the biocompatibility of the biodegradable materials, and the cost-effective manufacturing of these complex systems.

## 6. Conclusions

Wound healing is shifting from passive coverage to active intervention, aiming not merely to close wounds but to control the quality of healing. This review mainly summarizes the applications of biomaterials, including various wound dressings, cell-based strategies, and bioactive factor delivery systems, to reduce or inhibit scars and achieve ideal wound healing. Specifically, the integration of biomaterials that can reprogram the wound microenvironment, combined with the spatiotemporally controlled delivery of bioactive factors, represents a seminal advancement in the field. These systems move beyond the single-factor approaches and achieve scar-free wound healing, by orchestrating a sequential, multi-faceted healing response, that is, initially inhibiting excessive inflammation, then guiding fibroblast behavior towards regeneration rather than fibrosis, and, finally, promoting organized matrix remodeling.

Biomaterials have great potential in wound healing and scar treatment as research advances. However, there are still several challenges must be addressed to translate these technologies into clinical reality. Therefore, we propose the following priority areas for future research: Firstly, there is an urgent need to decipher the precise signaling cascades by which biomaterials influence immune cell polarity and fibroblast epigenetics to achieve regenerative outcomes. Secondly, future biomaterial systems should increasingly incorporate combinations of cytokines, miRNAs, and cells in “smart” scaffolds, which can respond to specific wound milieu cues (e.g., pH and enzyme levels) for on-demand therapy. Finally, large animal models can more accurately simulate human HTS, thus enabling rigorous evaluation of the long-term efficacy and safety of these sophisticated biomaterial strategies. Furthermore, whereas adult skin heals with a restorative scar, the fetus has the unique ability to regenerate entire layers of skin without scar tissue. In the future, breakthrough research achieved through comparison at the gene, molecular and cellular levels is expected to provide new ideas and methods for the scarless treatment and repair of human tissue damage.

## Figures and Tables

**Table 1:** Common methods of scar treatment at present.

Methods	Principle	Advantages	Disadvantages	Cost
Silicone-based Products (Gel/Sheets)	Forms a protective,	Non-invasive and safe. Easy to use.	Requires long-term, consistent use (months). Less effective for severe, established hypertrophic scars.	Low to medium cost.
Pressure Therapy	Applies continuous pressure, causing local tissue hypoxia, which flattens and softens the scar.	Effective for large-area scars, especially after burns.	Poor comfort; must be worn for long periods daily. Difficult to maintain effective pressure on joints or mobile areas.	Relatively high cost. Primarily the cost of custom-made pressure garments.
Corticosteroid Injections	Inhibits inflammatory response and collagen synthesis, softens, and flattens raised scars (hypertrophic scars and keloids).	Direct and effective for raised scars.	Requires multiple sessions. Potential side effects: skin atrophy, telangiectasia, hypopigmentation. Invasive and pain.	Medium cost per session.
Laser Therapy	Uses laser light for photothermal effects: stimulates collagen remodeling, ablates abnormal tissue, and improves scar color, texture, and thickness.	Minimally invasive with relatively quick recovery. Can be highly precise. Significant improvement in scar appearance.	Typically requires multiple treatment sessions. Risk of temporary redness, swelling, or hyper/hypopigmentation.	Relatively high cost.
Surgical Operation	Directly excises scar tissue and repairs the wound with fine suturing techniques or skin grafts/flaps.	Immediate effect for severe scars. Can correct functional impairments (e.g., contractures).	Invasive, with a longer recovery period. Risk of new scar formation. Usually requires combination with other therapies to prevent recurrence.	High cost.

**Table 3:** Dissimilarities in cell types, inflammatory levels, growth factors, and ECM components between adult and fetal wound healing.

Dissimilarities		Adult Wound Healing	Fetal Wound Healing
Inflammatory Response	Immune Cells	Abundant neutrophil infiltration; Predominantly pro-inflammatory M1 macrophages	Few neutrophils; Predominantly anti-inflammatory M2 macrophages and regulatory T cells
	Cytokine	High levels of IL-1β, PDGF, IL-6, and TNF-α	Low levels of IL-1β and IL-6; High levels of IL-10.
Cellular Phenotype & Activity	Fibroblasts	Slower proliferation; High propensity to differentiate into myofibroblasts	Rapid proliferation and migration; Minimal myofibroblast differentiation.
	Immune cell-fibroblast interaction	Powerful pro-inflammation and fibrosis interaction	Effective anti-inflammatory and regenerative interaction
ECM	Collagen	Predominantly Collagen I; Thick, disorganized fibers with high cross-linking	Higher Collagen III; Fine, reticular, and highly organized fibers
	Hyaluronic Acid	Transient early peak, rapidly degraded	Sustained high levels with abundant expression of HA receptors
Growth Factors	TGF-β Superfamily	High TGF-β1 and TGF-β2	High TGF-β3
	Fibroblast Growth Factors	Relatively lower levels	High levels, potently stimulating fibroblast proliferation and migration

## Data Availability

No new data were created or analyzed in this study. Data sharing is not applicable to this article.

## Abbreviations

The following abbreviations are used in this manuscript:

ECM	Extracellular matrix
MMPs	Matrix metalloproteases
α-SMA	Alpha-smooth muscle actin
TGF-β	Transforming growth factor-β
Fbs	Fibroblasts
CTGF	Connective tissue growth factor
EGF	Epidermal growth factor
VEGF	Vascular endothelial growth factor
ENF	En1-lineage-negative
EPF	Pro-fibrotic En1-positive
PDGF	Platelet-derived growth factor
HA	Hyaluronic acid
BC	Bacterial cellulose
SF	Silk fibroin
ADM	Acellular dermal matrices
PLCL	Poly(L-lactide-co-ε-capro-lactone)
HTS	Hypertrophic scar
PHBV	Poly(3-hydroxybutyrate-co-3-hydroxyvalerate)
PEGMA	Poly (ethylene glycol) methacrylate
bFGF	Basic fibroblast growth factor
IL-10	Interleukin-10
Rg3	Ginsenoside-Rg3
PDP	Pressure-driven permeation
TA	Triamcinolone acetonide
5-Fu	5-Fluorouracil
BMN	Bilayer dissolving microneedle
ZnS-NP	Zinc sulfide nanoparticles
ADSCs	Adipose-derived stem cells
MSCs	Mesenchymal stromal cells
GF	Graphene foam
BM-MSCs	Bone-marrow-derived mesenchymal stem cells
WJ-MSCs	Wharton’s jelly-derived mesenchymal stem cells
